# *In silico* identification of promising inhibitor against RNA-dependent RNA polymerase target of SARS-CoV-2 

**DOI:** 10.22099/mbrc.2021.40367.1621

**Published:** 2021-09

**Authors:** Pushpendra Singh, Manish Kumar Tripathi, Mohammad Yasir, Ruchi Khare, Rahul Shrivastava

**Affiliations:** 1 State Virus Research and Diagnostic Laboratory, Department of Microbiology, All India Institute of Medical Sciences, Raipur, Chhattisgarh-492099 India; 2 Department of Biophysics, All India Institute of Medical Sciences, New Delhi-110029, India; 3 Department of Nephrology, All India Institute of Medical Science Bhopal, Madhya Pradesh-462020 India; 4 Department of Biological Science and Engineering, Maulana Azad National Institute of Technology, Bhopal, Madhya Pradesh-462003 India; # Equally contributed, Pushpendra Singh and Manish Kumar Tripathi both are joint first author.

**Keywords:** SARS-CoV-2, COVID-19, RNA-dependent RNA polymerase (RdRp), In silico, Molecular Docking

## Abstract

The severe acute respiratory syndrome is a viral respiratory disease recognised as COVID-19, caused by severe acute respiratory syndrome coronavirus-2 (SARS-CoV-2). Formerly, no precise remedies are available, and many studies regarding COVID-19 prevention and treatment are under development. Several targets for the design of drugs are identified, and studies are in headway to explore the potential target. RNA-dependent RNA polymerase (RdRp) protein identified as a promising target against SARS-CoV-2 infection for the drug design due to its significant role in viral replication. The present study focuses on identifying the binding effect of previously known RdRp inhibitors with RdRp of SARS-CoV-2 using molecular docking and molecular dynamics simulation approaches. Molecular docking and binding free energy calculations against RdRp enzyme identified suramin as a potential compound that showed the highest docking score of -7.83 Kcal/mole and binding energy of -80.83 Kcal/mole as a comparison to other compounds. Further, molecular dynamics simulation studies were moreover showed the stable binding behaviour of suramin docked complex in the protein active site. Thus, the study concludes that suramin might be helpful as a potential inhibitor against RNA-dependent RNA polymerase of SRAS-CoV-2. However, further investigation is needed to assess the possible effect of inhibitors on RdRp through *in vitro* and *in vivo* experiments.

## INTRODUCTION

The new viral pneumonia cases were identified at the end of 2019; according to World Health Organization, coronavirus diseases-19 (COVID-19) is caused by a coronavirus which was named as 2019 novel coronavirus or “2019-nCoV” on January 12, 2020 [1]. Under international concern, WHO has termed it as ‘Pandemic’ and declared COVID-19 as the “*sixth public health emergency*” [2]. The origin of SARS related outbreak was marked by typical or atypical pneumonia disease at china in 2003 [3]. SARS are closely related to MERS-CoV (Middle East Respiratory Syndrome Coronavirus) as they share the same origin, i.e., both are zoonotic viruses, and of their similar host's, i.e. vertebrates such as humans, bat/civet and dromedary, respectively. Many studies showed that the RNA viral genome of coronavirus is a causative source of SARS. Thus, the disease was termed COVID-19 by WHO. SARS-CoV-2 is lethal to the human race because of its secure transmission among the population. The person infected with coronavirus showed symptoms such as; respiratory symptoms, cough, fever, shortness of breath, loss of smell and dyspnea, which may lead to pneumonia, and even death [4]. According to the world health organization, the world is at a high risk of this disease spread. Till 4^th^ April 2021, WHO has reported total 130,459,184 cases of positive cases in which 4,038,292 are new cases emerged in last 7 days. It has confirmed 2,842,325 deaths worldwide in which 71,355 death cases are reported in the previous 7 days [5]. 

The primary sources of SARS COVID-19 infection are animals, where transmission occurs from animal to humans, and then it spreads from human to human [6]. Different stages are classified amongst humans for transmitting this virus, such as interrupted transmissions, under investigation; imported cases only; local transmission, and community transmission. The transmission among the positive human patients shows different routes; the positive patients can be asymptomatic, pre-symptomatic and symptomatic [7]. The asymptomatic positive patients have a long incubation period within the humans where the carrier human is considered infectious, enabling transmission by respiratory droplets [8, 9]. Technically, people with the SARS-CoV-2 RNA virus are said to be positive. Coronaviruses are the largest group of positive-sense and single-stranded RNA viruses belonging to *Nidovirales* order, including Coronaviridae, Arteriviridae *Roniviridae* families [10]. Based on the phylogenetic clustering coronavirinae family segmented into four groups, the alpha, beta, gamma and delta coronaviruses, 2019-CoV belongs to a beta-coronavirus group [11]. According to cryo-electron microscopy and cryo-electron tomography studies, coronavirus is spherical shape with diameters of about 125 nm with club-like spike projections [12].

The coronavirus has a single-strand RNA genome that consists of a large conserved 30 Kb genome with three Open Reading Frames (ORFs) as ORF-1, ORF-2, and ORF-3, respectively [13, 14]. Structural genes in SARS-CoV-2 are encoded by the 3’end of RNA, it translate the four kinds of structural proteins such as: membrane (M), spike (S), nucleocapsid (N) and envelope (E), proteins. Spike (S) proteins contribute to the spike structures on the surface, act like fusion protein, and facilitate its attachment to the host cell receptors ACE2 [15-18]. Membrane (M) protein is the most abundant co-translationally inserted protein in the ER membrane and helps keep the virion structure intact [19]. The envelope (E) protein is responsible for the assembly and release of the virus [20]. Nucleocapsid (N) protein is present only in the nucleocapsid. As phosphorylation helps in RNA binding with another genomic RNA strand, this protein is found to be heavily phosphorylated, so it interacts with other protein domains present inside the virion and plays an significant role in subsequent packaging of RNA viral genome as a viral particle [21].

The key for SARS-CoV-2 replication is RNA-dependent RNA polymerase (RdRp) encoded at ORF-1 at 3’end with non-structural protein named nsp-12, which is also termed pol protein significant role in the replication process. The shape of RdRp is described as a right hand with palm, fingers, thumb, and N-terminal domain, which interact with thumb and finger domains. It has a greatly conserved active site located on the palm domain with motif A, motif B, motif C and motif D. Motif E is present in thumb domain, and the remaining two, motif F and motif G are present in finger domain [22]. There is a highly conserved step chain reactions occurred by the RNA-dependent RNA polymerases for viral genomic replication. The whole chain reaction machinery provides an efficient RNA polymerization activity through RdRp, including the proofreading and capping activities [23]. RdRp proved to be used as potential drug targets using various biochemical, cell line assays, proteomic and chemo-informatic analysis [24-27]. 

However, many strategies were applied to the identification of potential drugs for SARS-CoV-2. Here in the present study, we applied computational methods, namely molecular docking, MM-GBSA free energy calculation and molecular dynamics, to identify potential compound against the RdRp enzyme of COVID-19. 

## MATERIALS AND METHODS

**Identification of Ligand: **Based on the literature survey, we identify compounds that have potential antiviral effects (Supplementary Table 1). Further, compounds were sketched using 2d sketcher protocol, and Ligprep module was further used to prepare the ligands [28]. These optimized ligands were used for the computational studies. 

**Protein preparation and active site identification: **Molecular docking study was did with glide module of Schrödinger Maestro-2018-2 to identify the binding mode of prepared compounds with the crystal structure of RdRP (RNA dependent RNA polymerase) of COVID-19 (PDB ID: 6M71) [29]. The crystal structure of the RdRp protein was refined, pre-processed, minimized and optimized by using the protein preparation wizard module of Schrödinger Maestro 2018–2. The prime module was used to fill the missing or lost loops and side chains in the protein structure. The water molecules in the active site were removed, and the structure was optimized and minimized using the OPLS3 force field [30]. Finally, the prepared crystal structure was used to identify active sites using site map module of Schrödinger Maestro 2018-2 [31]. Site map identifies the binding sites using parameters such as size, enclosure, site score, exposure score, hydrophobic or hydrophilic character, donor or acceptor and contact. The receptor grid generation tool was applied to generate the grid near the identified binding site. Finally, a docking study was performed in the generated grid using prepared ligands acquired from the ligprep module. After docking glide score of docked compound was used to predict the binding affinity and results were visualized by using glide XP visualizer.

**Binding free energy calculation (MM-GBSA): **The binding free energy calculation is the widely used method in the computational drug designing process to envisage binding affinities in the protein-ligand complexes. MM-GBSA calculation was done by using the prime module of Schrodinger; in it, we input the pose viewer file of dock complex, and it provides an estimate of relative free energy of binding (∆G bind), which is calculated as given in the equation below:

MM/GBSA ΔG_bind_= G_complex_ - G_receptor_ - G_ligand_ Where G_complex_ - G_receptor_ - G_ligand_ represent the free energy of complex, receptor and ligand [32].

**Molecular dynamics simulation: **Further, the molecular dynamics (MD) simulation study was performed using Gromacs software to estimate the stability of the suramin compound in the active site of the RdRp enzyme [33]. Initially, the docked conformation of suramin got by docking was applied as preliminary conformation for the molecular docking study. The RdRp protein topology parameter was created by using GROMOS9643a1force filed, the topology file of surmain was made by using the PRODRG server [34]. Further, the complex was stayed in the cubic box with simple point charge (SPC216) water molecule and counter ions were added to neutralize the system. After neutralizing the solvated system, energy minimization was done firstly by steepest descent method followed by the conjugate gradient method to releasing the conflicting contact. Before the production run, the system (protein, counterions and ligands) was subjected to the position-restrained dynamics simulation (NVT and NPT) at 300 K for 100 ps to equilibrate the simulation system. The production run was performed for 50 ns simulation time at 300 K temperature and 1 bar pressure for 5,000 ps. Finally, after completing simulation, the trajectories were analysed for the simulation results.

## RESULTS AND DISCUSSION

COVID-19 cause by SARS-CoV-2, which belongs to a group of coronaviruses (CoVs) that infect humans [35, 36]. COVID-19 infections affect respiratory, digestive, liver, central nervous systems and patient with chronic disease [37]. The SARS-CoV-2 has a complex structure; many structural proteins are involved in virus entry into the host cells while non-structural proteins participate in the viral replication and into the suppression of the host immune system [4]. RNA-dependent RNA polymerase is a non-structural protein of SARS-CoV-2, which has a key role in the virus life cycle, especially in the viral RNA replication into the host cell cytoplasm. Due to these features, RdRp is widely considered a significant target against SRAS-CoV-2, and we use it as a target in the present study. It is also reported that the polymerase of COVID-19 has an overall 80% to 97% identity with the SARS polymerase [38]. Thus, the study is based upon assessing previously reported polymerase inhibition drugs to assess their effect targeting RdRp for COVID-19 treatment.

The selected inhibitors for the study are reported for *in silico* and *in vitro* RNA-dependent RNA polymerase inhibition within different RNA viruses. Site map tools are proved as a helpful tool for the identification of active sites, resulting in the generation of precise protein-ligand binding sites in the protein [39, 40]. The Dscore calculated by the site map provides an estimate of whether the site is druggable or not [41]. Thus, our calculated score showed that site1 showed the highest Dscore 1.043 (Best druggability score) (Table 1). The volume of the site1 pocket is also found to be 594.762 Å. Thus, based on DScore, Volume, size and site score, which was highest for site1, we selected site1 as a druggable pocked and generated the grid around site1 to perform the docking study for RdRp enzyme.

**Table 1:** Binding site identified using site map module

No	Site	Site Score	Size	Dscore	Volume
1	Site 1	1.022	196	1.043	594.762
2	Site 2	0.971	108	0.983	454.132
3	Site 3	0.998	108	0.983	454.132
4	Site 4	0.919	83	0.789	151.949
5	Site 5	0.968	82	0.796	242.158

**Figure 1:**
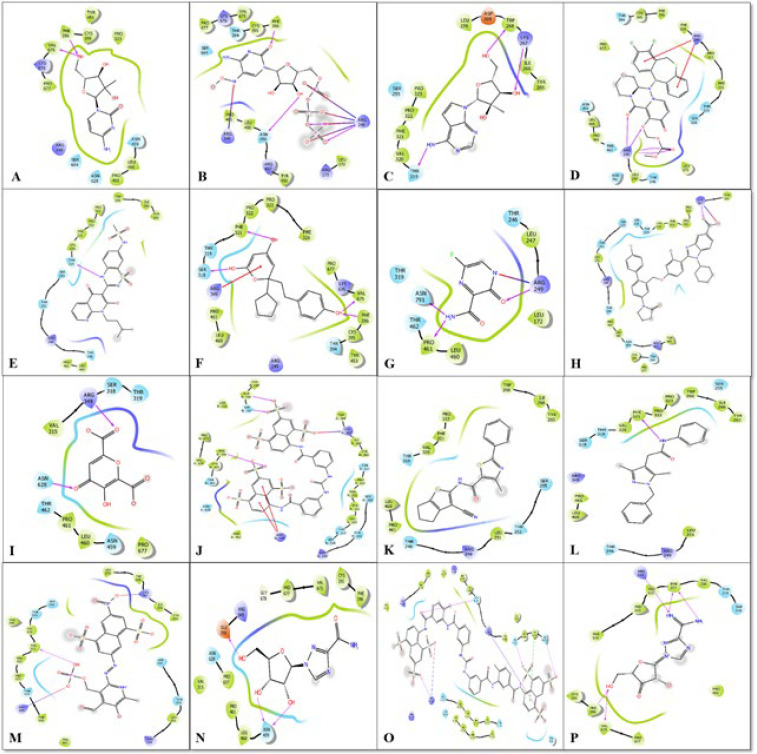
Docking pose analysis visualization of compounds: 2-C-Methylcytidine (A), 5-Niro Cytidine triphosphate (B), 7-Deaza-2'-C-methyladenosine (C), Baloxavir marboxil (D), Benzothiadiazines Compound 36 (E), Dihydropyrone Compound 53 (F), Favipiravir (G), JTK 109 (H), Meconic Acid (I), NF023 (J), NIC 02 (K), NIC 04 (L), PPNDS (M), Ribavirin (N), Suramin (O), Viramidine (P).

**Figure 2:**
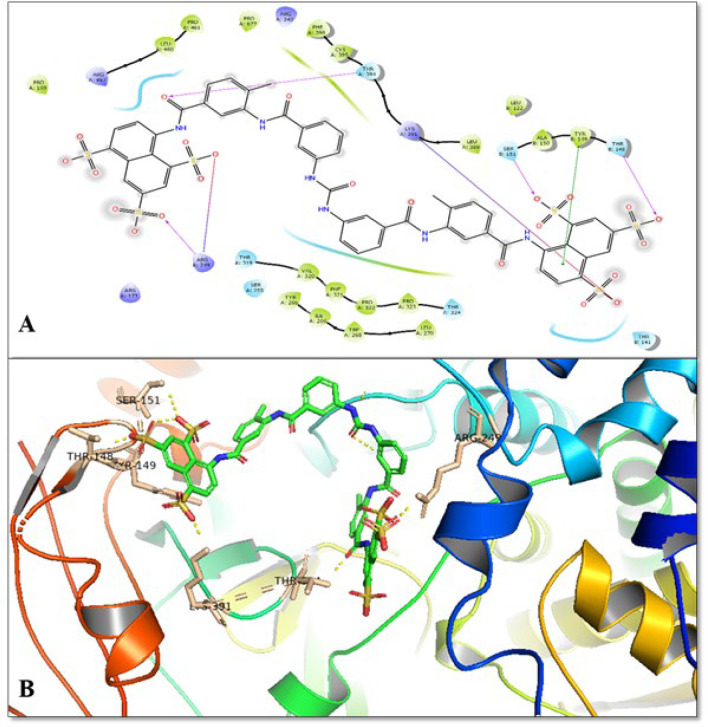
(A) 2-D docked pose interaction diagrammed of suramin (B) 3-D docked pose interaction diagrammed of suramin (Magenta colour) with RdRp enzyme

The selected small molecules were docked on the generated grid, which was predicted on the S1 binding site of RNA-dependent RNA polymerase to predict their potential against the target enzyme. The obtained results after the docking study were shown in Table 2, and their 2d interaction was shown in Figure 1. The results showed that the Glide score for all docked ligands was found to be in the range between -3.31 Kcal/mole to -7.83 Kcal/mole. The docking analysis showed that inhibition potential of sixteen compounds, ranked by glide score form high to low (ΔG) were found to be in the order; Suramin>5-Niro Cytidine triphosphate > F023 > Dihydropyrone Compound 53 > Ribavirin > NIC 04 > JTK 109 > Benzothiadiazines Compound 36>7-Deaza-2'-C-methyladenosine > Viramidine > 2-C-Methylcytidine > NIC 02 > Favipiravir > Meconic Acid > Baloxavir marboxil > PPNDS. Thus, according to the docking study, we identify suramin showed the best potential against RdRp enzyme compared to the other selected compounds. Suramin showed hydrogen bonding interaction with Thr-394, Arg-249, Ser-151, Thr-148 amino acid residues and π-π stacking interaction with Tyr-149 amino acid residues. Suramin was also found to be formed a salt bridge with Arg-249, Thr-394 and Lys-391 amino acid residues. The docking energies and interaction residues formed by the other selected compounds were shown in Table 2 and Figure 2

Further, MM-GBSA binding energies were calculated for all the docked complexes to estimate the relative binding affinities (ΔG binding energy) of all the docked compounds by using the prime module. The obtained results infer that MM-GBSA binding energies were found to be in the range of -4.19 kcal/mole to -80.83 kcal/mole. Suramin, which was found as potential compounds with the highest docking score -7.83 kcal/mole with protein RNA-dependent RNA polymerase, showed the highest binding energy -80.83 kcal/mol. 

Further, Molecular dynamics (MD) simulation study was done to evaluate the stability of suramin in the active site of the RdRp enzyme. The MD simulation study is widely used in computational drug designing to predict the stability of any drug molecule in the protein active site during the simulation time. The 30 ns simulation time was enough for the side-chain rearrangement and to gain the stable conformation of ligand in the protein's active site. After the production runs, the residual fluctuation in the protein and the stability of the docked complex was evaluated by using the root mean square fluctuation (RMSD) protocol (Fig. 2). The RMSD plot inferred that initially up to the 30 ns time scale, the fluctuation behaviour in the protein backbone and suramin dock complex was identified, and after this time scale, the RMSD graph showed stable behaviour with lesser fluctuations and was found to be in the acceptable region. Further, comparing the RMSD behaviour of RdRp protein and suramin, we found that suramin showed similar stable behaviour like the RdRp protein. 

**Table 2:** Docking results of the different compounds with RdRp enzyme of COVID-19

**No.**	**Compound Name**	**Glide Score**	**MM-GBSA** **(dG Binding Energy)**	** Interacting Residues**				
				**Hydrophobic Interaction**	**Polar Interaction**	**Hydrogen Bond**	**Pi-Pi Stacking**	**Pi Cation**
**1**	2-C-Methylcytidine	-5.04	-23.34	Pro-323, Cys-395, Phe-396, Leu-460, Pro-461, Tyr-453, Val-675, Pro-677	Asn -59, Asn-628, Ser-664	Phe-396, Val-675		
**2**	5-Niro Cytidine triphosphate	-6.69	-21.1	Leu-172, Cys-395, Phe-396, Tyr-456, Leu-460, Pro-461, Val-675, Pro-677	Thr-349, Asn,-459, Ser-664	Arg-249, Phe-396, Asn-459		
**3**	7-Deaza-2'-C-methyladenosine	-5.09	-36.29	Tyr-265, Ile-266, Trp-268, Leu-270, Val-320, Phe-321, Pro-322, Pro-323	Ser-255, Thr-319,	Lys-267, Trp-268, Thr-319		
**4**	Baloxavir marboxil	-3.75	-45.11	Leu-172, Leu-247, Phe-321, Pro-323, Phe-326, Cys-395, Phe-396, Leu-460, Pro-461, Pro-677	Thr-246, Ser-318, Thr-319, Thr-394, Asn-459, Thr-462, Asn-791	Arg-249		Arg-349
**5**	Benzothiadiazines Compound 36	-5.16	-42.75	Tyr-265, Ile-266, Trp-268, Val-320, Phe-321, Pro-322, Pro-323, Leu-460, Pro-461,	Thr-246, Thr-252, Ser-255, Thr-319	Thr-319		
**6**	Dihydropyrone Compound 53	-5.77	-41.82	Phe-321, Pro-322, Pro-323, Phe-326, Cys-395, Phe-396, Tyr-453, Leu-460, Pro-461, Val-675, Pro-677	Ser-318, Thr-319, Thr-394	Ser-318, Phe-321, Phe-396, Val-675		Ar-349
**7**	Favipiravir	-3.94	-10.20	Leu-172, Leu-247, Leu-460, Pro-461	Thr-246, Thr-319, Thr-462, Asn-791	Arg-249, Pro-461, Asn-791		
**8**	JTK 109	-5.19	-50.49	Leu-251, Tyr-265, Val-320, Phe-321, Pro-322, Pro-323, Cys-395, Tyr-456, Leu-460, Pro-461, Pro-677	Thr-246, Thr-252, Ser-255, Ser-318, Thr-319, Thr-394, Asn-459	Lys-267		
**9**	Meconic Acid	-3.9	-4.19	Val-315, Leu-460, Pro-461, Pro-677	Ser-318, Thr-319, Thr-462, Asn-459, Asn-628	Arg-349, Asn-628		
**10**	NF023	-6.06	-51.70	Tyr A-265, Ile A-266, Trp A-268, Val A-320, Phe A-321, Pro A-322, Pro A-323, Cys A-395, Phe A-396, Pro A-461, Val A-675, Pro A-677, Leu B-122, Tyr B-149, Ala B-150	Thr A-252, Ser A-255, Ser A-318, Thr A-319, Thr A-324, Thr A-349, Asn A-628, Ser B-151	Phe A-396, Ala B-150, Ser B-151		ArgA-349
**11**	NIC 02	-4.62	-51.98	Leu-251, Tyr-265, Ile-266, Trp-268, Val-320, Phe-321, Pro-322, Leu-460, Pro-461,	Thr-246, Thr-252, Ser-255, Thr-319			
**12**	NIC 04	-5.46	-48.67	Leu-251, Tyr-265, Ile-266, Trp-268, Val-320, Phe-321, Pro-322, Pro-323, Leu-460, Pro-461	Thr-246, Ser-255, Ser-318, Thr-319	Phe-321		
**13**	PPNDS	-3.31	-25.58	Leu-251, Tyr-265, Ile-266, Trp-268, Leu-270, Val-320, Phe-321, Pro-322, Pro-323, Phe-326, Phe-396, Pro-461,	Thr-252, Ser-255, Ser-318, Thr-319, Thr-324	Phe-321		
**14**	Ribavirin	-5.61	-23.27	Val-315, Cys-395, Phe-396, Leu-460, Pro-461, Pro-627, Val-675, Pro-677	Asn-459, Asn-628	Glu-350, Asn-459		
**15**	Suramin	-7.83	-80.21	Pro A-169, Tyr A-265, IleA-266, Trp A-268, Leu A-270, Phe A-321, Pro A-322, Pro A-323, Thr A-324, Leu A-389, Cys A-395, Phe A-396, Leu A-460, Pro A-461, Pro A-677, Leu B-122, Tyr B-149, Ala B-150	Ser A-255, Thr A-319, Thr A-349, Thr B-148, Ser B-151	Arg A-249, Thr A-394, Ser B-151, Thr B-148	Tyr B-149	
**16**	Viramidine	-5.07	-28.20	Val-320, Phe-321, Pro-322, Pro-323, Phe-326, Cys-395, Phe-396, Pro-461, Val-675, Pro-677	Ser-318, Thr-319	Phe-321, Arg-349, Phe-396, Val-675		

This stable behaviour of suramin showed that it was stabled in the active site of RdRp. The docking result of all the selected compound suramin was also confirmed the maximum negative dock score of -7.83 Kcal/mol. Similarly, the MM/GBSA binding energy of suramin was also found to be a maximum -80.21 Kcal/mol compared to the others.

**Figure 3:**
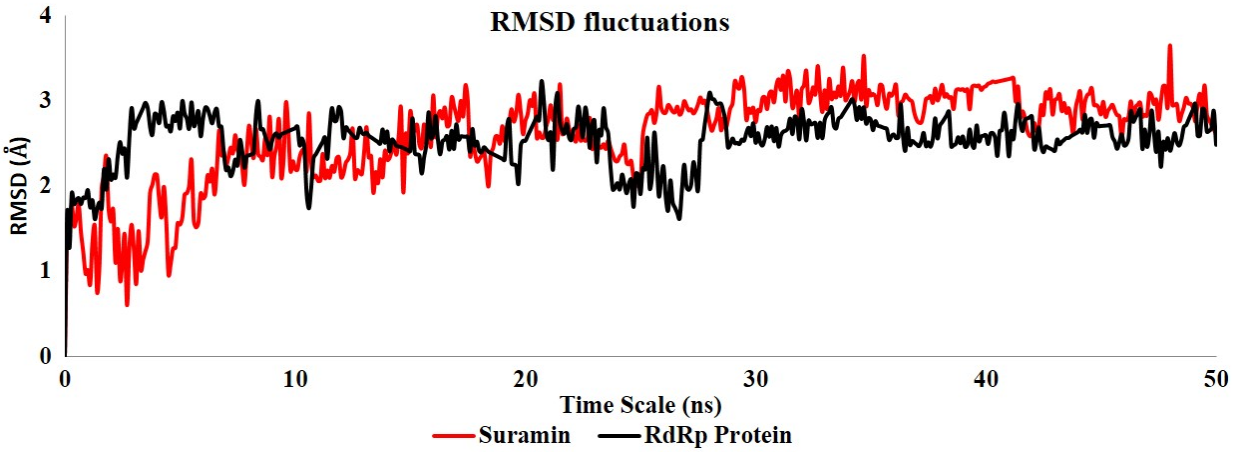
RMSD fluctuation of RdRp protein (Black) and suramin (Red) compound during 50 ns MD simulation

Our comprehensive study is based on the structure-based computational analysis to predict the interaction between RNA-dependent RNA polymerase of SARS-CoV-2 with different identified antiviral drugs. This target is the key player for viral replication and transcription of SARS-CoV-2. Currently, specific treatment for SARS-CoV-2 has not been reported despite ardent efforts. Thus, the use of the existing drug and conventional drugs are need for the present scenario to identify the potential drug against SARS-CoV-2. Although, some drugs are under clinical trials, and some are FDA approved, including Remdesivir (GS-5734) [26, 42, 43] . The computational approach is widely used to identify a potential drug molecule and open new possibilities for drug screening and development of COVID-19 treatment. Thus, the self-prepared library of potential antiviral compounds is made and docked in the target to identify the binding potential against the RdRp enzyme. We found that suramin showed its highest docking score (7.83 Kcal/mol) and binding energy (-80.21 Kcal/mol) compared to the other antiviral compounds from the proposed computational study. The RMSD trajectory of suramin was also found to be stable in the simulation period. Thus, this study concludes that suramin, which is widely used as an anticancer drug and reverse transcriptase inhibitor, showed their potential against the RdRp enzyme of SARS-CoV-2. 

## Supplementary Materials



## References

[1] WHO Novel Coronavirus (2019-nCoV) Sitution Report 22.

[2] Lai CC, Shih TP, Ko WC, Tang HJ, Hsueh PR (2020). Severe acute respiratory syndrome coronavirus 2 (SARS-CoV-2) and coronavirus disease-2019 (COVID-19): The epidemic and the challenges. Int J Antimicrob Agents.

[3] Hui DSC, Chan MCH, Wu AK, Ng PC (2004). Severe acute respiratory syndrome (SARS): epidemiology and clinical features. Postgrad Med J.

[4] Wu C, Liu Y, Yang Y, Zhang P, Zhong W, Wang Y, Wang Q, Xu Y, Li M, Li X, Zheng M, Chen L, Li H (2020). Analysis of therapeutic targets for SARS-CoV-2 and discovery of potential drugs by computational methods. Acta Pharm Sin B.

[5] WHO Weekly epidemiological update on COVID-19-6 April 2021.

[6] Chan JFW, Yuan S, Kok KH, To KKW, Chu H, Yang J, Xiang F, Liu J, Yip C, Shan-Poon RW, Tsoi HW, Kam-Fai Lo S, Chan KH, Poon VKM, Chan WM, Daniel J, Cai JP, Chung-Cheng VC, Chen H, Ming-Hui CK, Yuen, KY (2020). A familial cluster of pneumonia associated with the 2019 novel coronavirus indicating person-to-person transmission: a study of a family cluster. Lancet.

[7] WHO Coronavirus disease 2019 (COVID-19) Situation Report–73.

[8] Kimball A, Hatfield K, Arons M, James A, Taylor J, Spicer K, Bardossy AC, Oakley LP, Tanwar S, Chisty Z, Bell JM, Methner M, Harney J, Jacobs JR, Carlson CM, Mclaughlin HP, Stone N, Clark S, Smith CB, Page LC, Kay M, Lewis J, Russell D, Hiatt B, Gant J, Duchin JS, Clark TA, Honein MA, Reddy SC, Jernigan JA, Baer A, Barnard L, Benoliel E, Fagalde MS, Ferro J, Smith HG, Gonzales E, Hatley N, Hatt G, Hope M, Frazier MH, Kawakami V, Lenahan JL, Lukoff MD, Mier EB, Mckeiranan S, Montgomery P, Morgan JL, Mummert LA, Pogosjanse S, Riedo F, Schwarcz L, Smith D, Stearns S, Sykes KJ, Whitney H, Ali H, Banks M, Balajee A, Chow E, Cooper B, Currie D, Dyal J, Healy J, Hughes M, Mcmichael T, Nolen L, Olson C, Rao A, Schmit K, Schwartz N, Tobolowsky F, Zacks R, Zane SB (2020). Asymptomatic and presymptomatic SARS-CoV-2 infections in residents of a long-term care skilled nursing facility- King County, Washington, March 2020. MMWR Morb Mortal Wkly Rep.

[9] Rothe C, Schunk M, Sothmann P, Bretzel G, Froeschl G, Wallrauch C, Zimmer T, Thiel V, Janke C, Guggemos W, Seilmaier M, Drosten C, Vollmar P, Zwirglmaier K, Zange S, Wolfel R, Hoelscher M (2020). Transmission of 2019-nCoV infection from an asymptomatic contact in Germany. N Engl J Med.

[10] Fehr AR, Perlman S (2015). Coronaviruses: an overview of their replication and pathogenesis. Methods Mol Biol.

[11] Zumla A, Chan JFW, Azhar EI, Hui DSC, Yuen KY (2016). Coronaviruses - drug discovery and therapeutic options. Nat Rev Drug Discov.

[12] Bárcena M, Oostergetel GT, Bartelink W, Faas FGA, Verkleij A, Rottier PJM, Koster AJ, Bosch BJ (2009). Cryo-electron tomography of mouse hepatitis virus: Insights into the structure of the coronavirion. Proc Natl Acad Sci U S A.

[13] Clarke IN, Lambden PR (2000). Organization and expression of calicivirus genes. J Infect Dis..

[14] Singh P, Tripathi MK, Yasir M, Khare R, Tripathi MK, Shrivastava R (2020). Potential inhibitors for SARS-CoV-2 and functional food components as nutritional supplement for COVID-19: A review. Plant Foods Hum Nutr.

[15] de Groot RJ, Luytjes W, Horzinek MC, van der Zeijst BAM, Spaan WJM, Lenstra JA (1987). Evidence for a coiled-coil structure in the spike proteins of coronaviruses. J Mol Biol.

[16] Bosch BJ, Zee RVD, Haan CAM, Rottier PJM (2003). The coronavirus spike protein is a class I virus fusion protein: structural and functional characterization of the fusion core complex. J Virol.

[17] Tripathi MK, Singh P, Sharma S, Singh TP, Ethayathulla AS, Kaur P (2020). Identification of bioactive molecule from Withania somnifera (Ashwagandha) as SARS-CoV-2 main protease inhibitor. J Biomol Struct Dyn.

[18] Singh P, Tripathi KM, Shrivastava R (2021). In silico identification of linear B-cell epitope in Coronavirus 2019 (SARS-CoV-2) surface glycoprotein: a prospective towards peptide vaccine. Minerva Biotechnol Biomol Res.

[19] Neuman BW, Kiss G, Kunding AH, Bhella D, Baksh MF, Connelly S, Droese B, Klaus JP, Makino S, Sawicki SG, Siddell SG, Stamou DG, Wilson IA, Kuhn P, Buchmeier MJ (2011). A structural analysis of M protein in coronavirus assembly and morphology. J Struct Biol.

[20] Nieto-Torres JL, DeDiego ML, Verdiá-Báguena C, Jimenez-Guardeño JM, Regla-Nava JA, Fernandez-Delgado R, Castano-Rodriguez C, Alcaraz A, Torres J, Aguilella VM, Enjuanes L (2014). Severe acute respiratory syndrome coronavirus envelope protein ion channel activity promotes virus fitness and pathogenesis. PLoS Pathog.

[21] Chang C, Sue SC, Yu T, Hsieh CM, Tsai CK, Chiang YC, Lee SJ, Hsiao HH, Wu WJ, Chang WL, Lin CH, Huang TH (2006). Modular organization of SARS coronavirus nucleocapsid protein. J Biomed Sci.

[22] Deval J, Jin Z, Chuang YC, Kao CC (2017). Structure(s), function(s), and inhibition of the RNA-dependent RNA polymerase of noroviruses. Virus Res.

[23] Subissi L, Posthuma CC, Collet A, Zevenhoven-Dobbe JC, Gorbalenya AE, Decroly E, Snijder EJ, Canard B, Imbert I (2014). One severe acute respiratory syndrome coronavirus protein complex integrates processive RNA polymerase and exonuclease activities. Proc Natl Acad Sci USA.

[24] Ju J, Li X, Kumar S, Jockusch S, Chien M, Tao C, Morozova I, Kalachikov S, Kirchdoerfer RN, Russo JJ (2020). Nucleotide analogues as inhibitors of SARS-CoV polymerase. Pharmacol Res Perspect.

[25] Xue X, Yu H, Yang H, Xue F, Wu Z, Shen W, Li J, Zhou Z, Ding Y, Zhao Q, Zhang XC, Liao M, Bartlam M, Rao Z (2008). Structures of two coronavirus main proteases: implications for substrate binding and antiviral drug design. J Virol.

[26] Wang M, Cao R, Zhang L, Yang X, Liu J, Xu M, Shi Z, Hu Z, Zhong W, Xiao G (2020). Remdesivir and chloroquine effectively inhibit the recently emerged novel coronavirus (2019-nCoV) in vitro. Cell Res.

[27] Wu YS, Lin WH, Hsu JTA, Hsieh HP (2006). Antiviral drug discovery against SARS-CoV. Curr Med Chem.

[28] Schrödinger (2018). Release 2018-2: LigPrep.

[29] Gao Y, Yan L, Huang Y, Liu F, Zhao Y, Cao L, Wang T, Sun Q, Ming Z, Zhasng L, Ge J, Zheng L, Zhang Y, Wang H, Zhu Y, Zhu C, Hu T, Hua T, Zhang B, Yang X, Li J, Yang H, Liu Z, Xu W, Guddat LW, Wang Q, Lou Z, Rao Z (2020). Structure of the RNA-dependent RNA polymerase from COVID-19 virus. Science.

[30] Jorgensen WL, Maxwell DS, Tirado-Rives J (1996). Development and testing of the OPLS all-atom force field on conformational energetics and properties of organic liquids. J Am Chem Soc.

[31] Schrödinger (2018). Release 2018-2: Sitemap.

[32] Jacobson MP, Pincus DL, Rapp CS, Day TJF, Honig B, Shaw DE, Friesner RA (2004). A hierarchical approach to all-atom protein loop prediction. Proteins.

[33] Bowers KJ, Chow DE, Xu H, Dror RO, Eastwood MP, Gregersen BA, Klepeis JL, Kolossvary I, Moraes MA, Sacerdoti FD, Salmon JK, Shan Y, Shaw DE (2006). Scalable Algorithms for Molecular Dynamics Simulations on Commodity Clusters. SC’06 Proc.

[34] Kaczor AA, Targowska-Duda KM, Patel JZ, Laitinen T, Parkkari T, Adams Y, Nevalainen TJ, Poso A (2015). Comparative molecular field analysis and molecular dynamics studies of α/β hydrolase domain containing 6 (ABHD6) inhibitors. J Mol Model.

[35] Li H, Liu SM, Yu XH, Tang SL, Tang CK (2020). Coronavirus disease 2019 (COVID-19): current status and future perspectives. Int J Antimicrob Agents.

[36] Saini S, Saini A, Thakur CJ, Kumar V, Gupta RD, Sharma JK (2020). Genome-wide computational prediction of miRNAs in severe acute respiratory syndrome coronavirus 2 (SARS-CoV-2) revealed target genes involved in pulmonary vasculature and antiviral innate immunity. Mol Biol Res Commun.

[37] Halpin DMG, Faner R, Sibila O, Badia JR, Agusti A (2020). Do chronic respiratory diseases or their treatment affect the risk of SARS-CoV-2 infection?. Lancet Respir Med.

[38] Elfiky AA (2020). Anti-HCV, nucleotide inhibitors, repurposing against COVID-19. Life Sci.

[39] Tripathi MK, Yasir M, Singh P, Shrivastava R (2020). A comparative study to explore the effect of different compounds in immune proteins of human beings against tuberculosis: Insight from docking and molecular dynamics studies. Curr Bioinform.

[40] Shrivastava R, Yasir M, Tripathi M, Singh P (2016). In silico interaction of methyl isocyanate with immune protein responsible for Mycobacterium tuberculosis infection using molecular docking. Toxicol Ind Health.

[41] Halgren TA (2009). Identifying and characterizing binding sites and assessing druggability. J Chem Inf Model.

[42] Tripathi MK, Sharma S, Singh T, Ethayathulla AS, Kaur P (2021). Computational intelligence in drug repurposing for COVID-19 Comput intell methods COVID-19 surveillance. Prev Predict Diagnosis Stud Comput Intell.

[43] Davies M, Osborne V, Lane S, Roy D, Dhanda S, Evans A, Shakir S (2020). Remdesivir in treatment of COVID-19: A systematic benefit-risk assessment. Drug Saf.

