# Supplementary material for: In silico identification of promising inhibitor against RNA-dependent RNA polymerase target of SARS-CoV-2
Source: Mol Biol Res Commun. 2021 Sep;10(3):131–40. doi: 10.22099/mbrc.2021.40367.1621 (PMC8340315; doi:10.22099/mbrc.2021.40367.1621)
Supplement: Supplementary file 1 [file mbrc-10-131-s001.pdf]

Table S1: *2D Structure of the selected compounds*

S. No.	Compound Name	Structure	References
**1.**	2-C-Methylcytidine	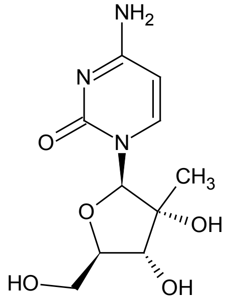	[1]
**2.**	5-Niro Cytidine triphosphate	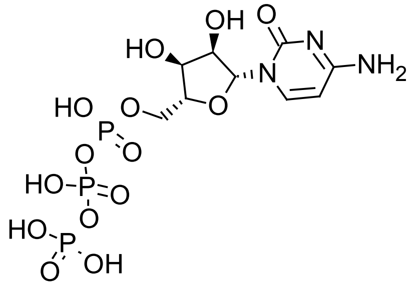	[2, 3]
**3.**	7-Deaza-2'-C-methyladenosine	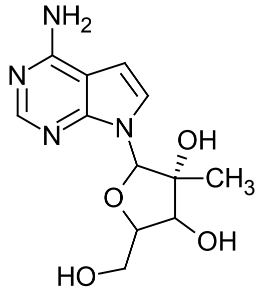	[4]
**4.**	Baloxavir marboxil	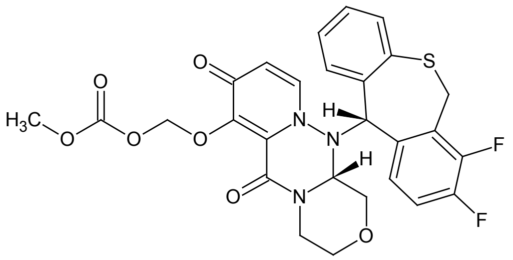	[5]

**5.**	Benzothiadiazines<br>Compound 36	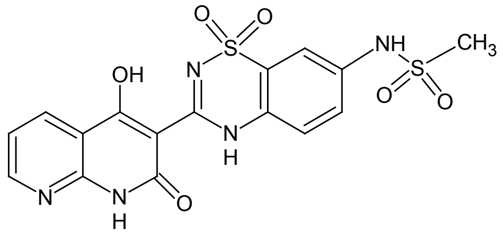	[6]
**6.**	Dihydropyrone<br>Compound 53	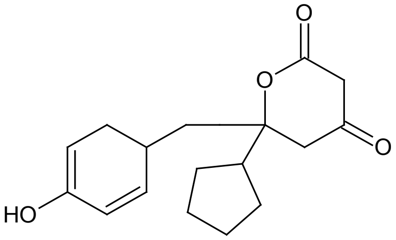	[7]
**7.**	Favipiravir	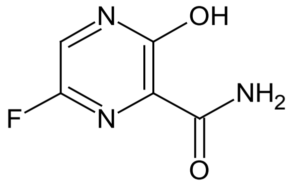	[8]
**8.**	JTK 109	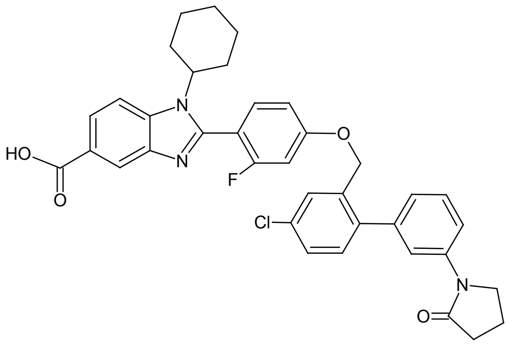	[9, 10]
**9.**	Meconic Acid	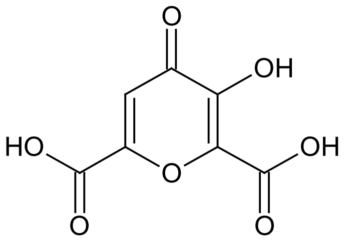	[11]

10. NF023 [12]

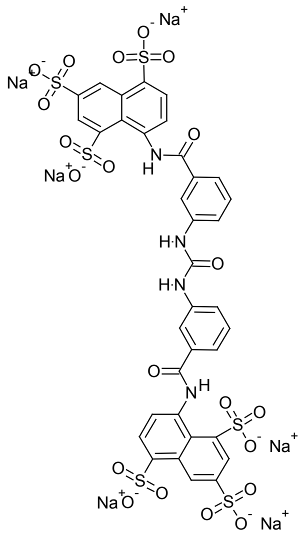

11. NIC 02 [13]

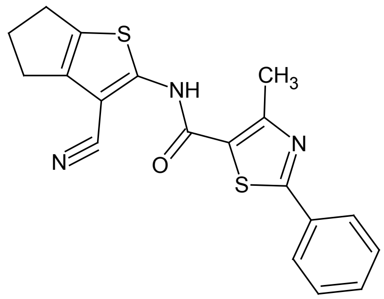

12. NIC 04 [13]

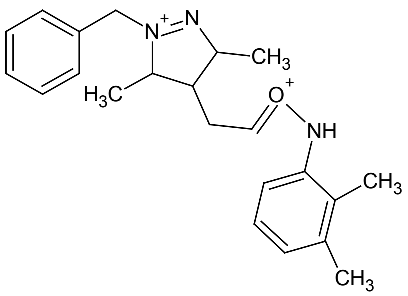

13. PPNDS [14]

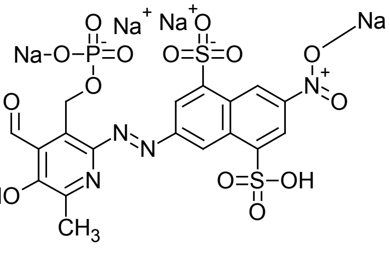

14. Ribavirin [15]

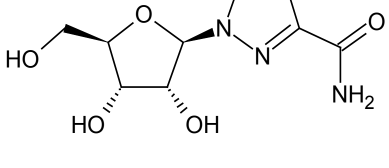

15. Suramin 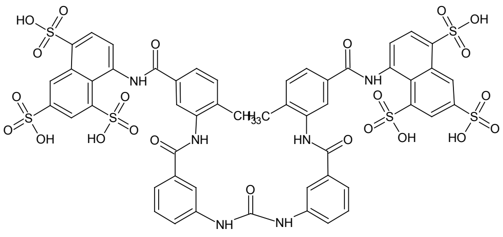 [16]

16. Viramidine 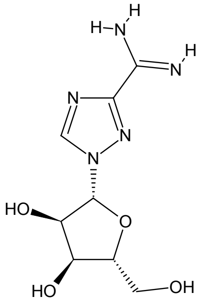 [17]

REFERENCES

1. Rocha-Pereira J, Jochmans D, Debing Y, Verbeken E, Nascimento MSJ, Neyts J. The viral polymerase inhibitor 2'-C-methylcytidine inhibits Norwalk virus replication and protects against norovirus-induced diarrhea and mortality in a mouse model. J Virol 2013;87:11798-11805.
2. Deval J, Jin Z, Chuang YC, Kao CC. Structure(s), function(s), and inhibition of the RNA-dependent RNA polymerase of noroviruses. Virus Res 2017;234:21-33.
3. Harki DA, Graci JD, Galarraga JE, Chain WJ, Cameron CE, Peterson BR. Synthesis and antiviral activity of 5-substituted cytidine analogues: identification of a potent inhibitor of viral RNA-dependent RNA polymerases. J Med Chem 2006;49:6166-6169.
4. Eyer L, Fojtíková M, Nencka R, Rudolf I, Hubálek Z, Ruzek D. Viral RNA-dependent RNA polymerase inhibitor 7-Deaza-2'- C-methyladenosine prevents death in a mouse model of West Nile virus infection. Antimicrob Agents Chemother 2019;63:e02093-e03018.
5. Omoto S, Speranzini V, Hashimoto T, Noshi T, Yamaguchi H, Kawai M, Kawaguchi K, Uehara T, Shishido T, Naito A, Cusack S. Characterization of influenza virus variants induced by treatment with the endonuclease inhibitor baloxavir marboxil. Sci Rep 2018; 8:9633.
6. Rockway TW, Zhang R, Liu D, Betebenner DA, McDaniel KF, Pratt JK, Beno D, Montgomery D, Jiang WW, Masse S, Kati WM, Middleton T, Molla A, Maring CJ, Kempf DJ. Inhibitors of HCV NS5B polymerase: Synthesis and structure–activity relationships of N-1-benzyl and N-1-[3-methylbutyl]-4-hydroxy-1,8-naphthyridon-3-yl benzothiadiazine analogs containing substituents on the aromatic ring. Bioorg Med Chem Lett 2006;16:3833-3838.
7. Li H, Tatlock J, Linton A, Gonzalez J, Borchardt A, Dragovich P, Jewell T, Prins T, Zhou R, Blazel J, Parge H, Love R, Hickey M, Doan C, Shi S, Duggal R, Lewis C, Fuhrman S. Identification and structure-based optimization of novel dihydropyrones as potent HCV RNA polymerase inhibitors. Bioorg Med Chem Lett 2006;16:4834-4838.
8. Furuta Y, Gowen BB, Takahashi K, Shiraki K, Smee DF, Barnard DL. Favipiravir (T-705), a novel viral RNA polymerase inhibitor. Antiviral Res 2013;100:446-454.
9. Hirashima S, Oka T, Ikegashira K, Noji S, Yamanaka H, Hara Y, Goto H, Mizojiri R, Niwa Y, Noguchi T, Ando I, Ikeda S, Hashimoto H. Further studies on hepatitis C virus NS5B RNA-dependent RNA polymerase inhibitors toward improved replicon cell activities: Benzimidazole and structurally related compounds bearing the 2-morpholinophenyl moiety. Bioorg Med Chem Lett 2007;17:3181-186.

10. Hirashima S, Suzuki T, Ishida T, Noji S, Yata S, Ando I, Komatsu M, Ikeda S, Hashimoto H. Benzimidazole derivatives bearing substituted biphenyls as hepatitis C virus NS5B RNA-dependent RNA polymerase inhibitors: Structure−activity relationship studies and identification of a potent and highly selective inhibitor JTK-109. J Med Chem 2006;49: 4721-4736.
11. Pace P, Nizi E, Pacini B, Pesci S, Matassa V, De Francesco R, Altamura S, Summa V. The monoethyl ester of meconic acid is an active site inhibitor of HCV NS5B RNA-dependent RNA polymerase. Bioorg Med Chem Lett 2004;14:3257-3261.
12. Tarantino D, Pezzullo M, Mastrangelo E, Croci R, Rohayem J, Robel I, Bolognesi M, Milani M. Naphthalene-sulfonate inhibitors of human norovirus RNA-dependent RNA-polymerase. Antiviral Res 2014;102:23-28.
13. Eltahla AA, Lim KL, Eden JS, Kelly AG, Mackenzie JM, White PA. Nonnucleoside inhibitors of Norovirus RNA polymerase: Scaffolds for rational drug design. Antimicrob Agents Chemother 2014;58:3115-3123.
14. Croci R, Tarantino D, Milani M, Pezzullo M, Rohayem J, Bolognesi M, Mastrangelo E. PPNDS inhibits murine Norovirus RNA-dependent RNA-polymerase mimicking two RNA stacking bases. FEBS Lett 2014;588:1720-1725.
15. Mirza MU, Vanmeert M, Froeyen M, Ali A, Rafique S, Idrees M. In silico structural elucidation of RNA-dependent RNA polymerase towards the identification of potential Crimean-Congo Hemorrhagic Fever Virus inhibitors. Sci Rep 2019;9:6809.
16. Croci R, Pezzullo M, Tarantino D, Milani M, Tsay SC, Sureshbabu R, Tsai YJ, Mastrangelo E, Rohayem J, Bolognesi M, Hwu JR. Structural bases of norovirus RNA dependent RNA polymerase inhibition by novel suramin-related compounds. PLoS One 2014;9:e91765.
17. Chern RRD, JW NS5B. RNA dependent RNA polymerase inhibitors: The promising approach to treat hepatitis C virus infections. Curr Med Chem 2010;3806-3826.
